# More Maternity Hospitals Wanted

**Published:** 1920-05-29

**Authors:** 


### MORE MATERNITY HOSPITALS WANTED.

The Medical Officer of Health for York (Dr. E. M. Smith) addressed a conference of the Yorkshire Branch of the Society of Medical Officers of Health at Scarborough, and took as his subject the *rôle* of the maternity hospital in public health work. Giving the figures of maternal mortality in England and Wales in 1918 as one for every 243 births —or 1,239 deaths for the year—he asserted that an enormous amount of maternal mortality and disease could be prevented; this is incontrovertible. Progress has been made in rapid strides, and Dr. Smith attributes it to improved midwifery and institutional treatment, especially in London, where good institutions are numerous and where maternal mortality has diminished more than in the provinces. Dr. Smith strongly advocates institutional care of maternity cases because of the life- and health-saving value, but the shortage of houses is responsible for the increased demand upon the already limited accommodation now existing. Mothers also have realised the value of the peacefulness and skilled attention of the maternity homes and hospitals. The demand for maternity accommodation among the more opulent classes has been very remarkable during the last two or three years.

Dr. Smith hopes to see a great extension of present facilities, which would lessen the dangers of confinement in small, overcrowded, and often unhealthy homes. But maternity hospitals are rare, and, so far as he knew, there are only thirteen in provincial England and Wales managed and financed by the municipality; these are nearly all small. He hopes further extensions will be made as self-supporting as possible.

